# Development of a Multisensor-Based Bio-Botanic Robot and Its Implementation Using a Self-Designed Embedded Board

**DOI:** 10.3390/s111211629

**Published:** 2011-12-13

**Authors:** Chung-Liang Chang, Ming-Fong Sie, Jin-Long Shie

**Affiliations:** Department of Biomechatronics Engineering, National Pingtung University of Science and Technology, No. 1 Shuefu Road, Neipu, Pingtung County 91201, Taiwan; E-Mails: m9844014@mail.npust.edu.tw (M.-F.S.); j357753k@yahoo.com.tw (J.-L.S.)

**Keywords:** embedded system, bio-botanic robot, micro-controller

## Abstract

This paper presents the design concept of a bio-botanic robot which demonstrates its behavior based on plant growth. Besides, it can reflect the different phases of plant growth depending on the proportional amounts of light, temperature and water. The mechanism design is made up of a processed aluminum base, spring, polydimethylsiloxane (PDMS) and actuator to constitute the plant base and plant body. The control system consists of two micro-controllers and a self-designed embedded development board where the main controller transmits the values of the environmental sensing module within the embedded board to a sub-controller. The sub-controller determines the growth stage, growth height, and time and transmits its decision value to the main controller. Finally, based on the data transmitted by the sub-controller, the main controller controls the growth phase of the bio-botanic robot using a servo motor and leaf actuator. The research result not only helps children realize the variation of plant growth but also is entertainment-educational through its demonstration of the growth process of the bio-botanic robot in a short time.

## 1. Introduction

Recently, owing to the advancement in digitalized life technology and promotion of Micro-Electro Mechanical Systems (MEMS) technology, the robotics industry has developed rapidly and moved closer to the improvement of human life quality and demand, among which bio-robots have become a popular topic recently. Besides the interactive amusement that bio-robot systems bring to people, the systems can serve as vehicles to aid people. Currently, amusement robots enjoy great popularity. The amusement bio-robots available on the market are not only equipped with lively and lovely body language but are also capable of interacting with people, which subverts people’s view about the inactive traditional toys [1–5].

The amusement bio-robots on the market are various, and consist of maritime class, terrestrial and birds. Plants occupy two categories among the three. Surprisingly, related products available in market are quite rare despite the fact that plants are so essential and common in life. Edible plants can adjust our diet and physical condition and ornamental plants can soothe people, soften the atmosphere and condition the air.

Many cases of bionic system development have taken place. For example, the website of The Center of Biologically Inspired Design at the Georgia Institute of Technology has offered prolific research results on bionics [6], and the Department of Mechanical Engineering of the University of Maryland and the University of Reading are both engaged in the development and research of bionic design products, with whole websites presenting abundant research results [7,8].

Among the design methods of bionic system, the website of the Biomimicry Institute has provided the design concept, process, bionic methods, and has classified those cases [9]. The classification consists of material, product, architecture, and process. The design spiral concept is proposed and the concept includes several steps, such as identify, interpret, discover, abstract, emulate, and evaluate. Vakili and Shu propose a method to analyze the problem in the design of bionics [10].

Chiu and Shu published several papers utilizing the analysis of natural language, which systematically employs engineering to mimic the natural behavior of creatures [11–13]. The birth of bionic design originates from the fact that human technology development encounters bottlenecks and is accidentally inspired by the behavior characteristics of creatures in the search for solutions in Nature. Thus, bionics are generated and this design has been termed Bio-Inspired Design by scientists. Many famous products come from such a design, such as shark swimsuit, water pump, architecture design, *etc*. Therefore, Golden classified the function and concept of Bio-Inspired Design and established a database so that engineers are provided a solution from the design concepts and function [14]. Afterwards, lots of literature reviews have also focused on the design and control of intelligent mechanisms [15–18]. Most of them utilize some intelligent algorithms to simulate the behavior of creatures and adopt automatic mechanisms to implement those gestures and behavior.

No product is currently available on market that can mimic the behavior of plants. The solar flower product developed in Japan is simple in function and is not interactive [19]. In view of this, this research mimics the growth model of plants through bionics and proposes a multisensor-based bio-botanic robot.

With regard to the structure design of the robot, the root design adopts an aluminum screw base. The pulley and spring are employed to mimic the growth and bending features of plant bodies. Polydimethylsiloxane (PDMS) is adopted as the material of leaves and petals. The swing of leaves and flowing of petals is accomplished using shape memory alloy (SMA) and self-designed actuator. In terms of design of control system, this paper aims to regulate the growth speed and behavior of plants using multi-staged fuzzy logic scheme on the basis of light, water, and temperature in an experimental environment through expert data. The result is implemented in the robot system. The environment sensor module, SMA actuator and motor control device are integrated in the self-designed sensor-fusion based embedded board. After the environment sensor data is processed through the micro-controller algorithm, the micro-controller sends different control signals to the motor control and actuator module of the embedded board so that the robot can present different growth behaviors.

The development of bio-botanic robot system (BRPS) aim to balance body and soul, usher people to a digitalized green life and create an environment in harmony with Nature. The following chapter will depict the design process of robot and the test results. Meanwhile, it takes a longer time to teach children the growth process of plants. Through the establishment of bio-robots, children can experience such a process within a shorter time by means of the rapid and dynamic response of plants. On the other hand, the bio-botanic robot serves as an experimental tool of automatic cultivation through the establishment of an environmental database.

## 2. Overall Design of the Bio-Botanic Robot

### 2.1. Planning of the Bio-Botanic Robot Growth System

The sunflower is an extraordinary and magical flower, whose uplifting stance is associated with aspiration, the big face embracing the sunshine is indicative of positive thinking for humans and the downward flower due to the weight of mature seeds is a reminder of maturity and humility. More significantly, it can be used for ornamental gardening, agriculture, as an economic plants and for spiritual therapy. Based on those factors, this paper takes sunflower as an example to implement a bionic plant mechanism according to its growth characteristics. Before the design and setup of the BRPS, it is necessary to be familiar with the characteristics and mechanism of plants.

This research adopted sunflowers as the subject for growth simulation due to its wide applicability as a source of food, oil, animal feed, and biofuel. In accordance with the literature, the growth process of sunflowers was divided into eight stages that mark the approximate range of time required for each stage [20–24]. To simplify the analysis and facilitate the design of the growth regulation system, this study divided the plant growth process into stages according to the number of environmental factors influencing growth height and growth time. In previous research, the authors presented the environmental factors required by sunflowers in each stage of growth as well as the corresponding growth height and growth time [25]. The authors also developed a multi-layer parallel fuzzy inference system with the objective of establishing an expert system to monitor plant growth. The steps involved in designing the BRPS are as follows:

Step 1: Based on the eight growth stages identified in this study, we used the proportions of individual environmental factors obtained from literature and the experience of experts to establish environmental parameters suitable for each stage of growth.
Step 2: We produced a bionic botanical growth fuzzy controller comprising a fuzzy main-controller and sub-controllers.
Step 3: The results obtained by the fuzzy controller were compared with data from the literature.

In general, fuzzy controllers are designed in the following order: (1) Definition of input variables, output variables, and linguistic variable database; (2) Determination of fuzzification strategies; (3) Design of controlling rule database; (4) Develop methods of fuzzy inference; (5) Defuzzification. As seen in [25], environmental factors indeed influence plant growth height and growth time, even without considering the generic characteristics of the plant itself. This research divided the plant growth process into stages according to the number of environmental factors influencing growth height and growth time. Excluding the wither stage, we established seven stages numbered from 0 to 6. This research utilizes the previously proposed control method to be implemented in an embedded development system. Based on the above description, we will define the mechanism and control component that constitute the bionic mechanism and system.

### 2.2. Architecture and Feature of Bio-Botanic Robot

This chapter describes the bio-botanic robot design process. The use of a small size robot base, flower institutions, and control motor with linearity are the main concepts in this study. Meanwhile, the body curvature of plant can not only move up and down but also to make curved, swinging action. The system design steps are as follows:

#### 2.2.1. Plant Root

**Figure 1.**
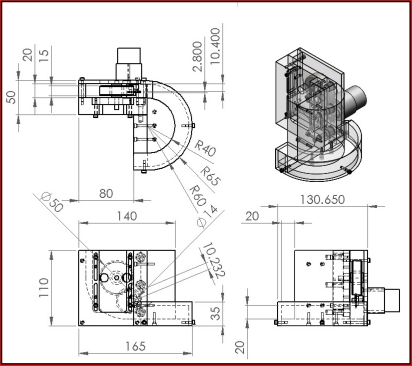
The 3D view of the growth base.

The root of plant can move up and down and its goal is to represent the growth process. Firstly, the plant is contained in the flowerpot. The transmission concept is based on that between gear and rack and the spring is employed as a substitute for the rack. The spring is better suited to match the gear because the spring is shaped in the form of a rack pitch and can bite perfectly with gear. The design is shown in Figure 1.

#### 2.2.2. Plant Stem

The plant stem should present ductility, curvature and swinging function to sustain leaves and flowers so that the plant appears more vivid. Thus, the spring is adopted to substitute stem part because the spring can also present ductility and curvature like the plant itself. The spring at the bottom is fixed and its top point is pressed, which allows the spring to present curvature movement. It is like the natural vertical drop of sunflower leaves.

The parameter setup of the spring is based on the actual shape and weight of a real sunflower to give setup values of different ratio. The diameter of sunflower stems vary according to their variety and is typically about 10∼25 mm. The height of plants is 1.5∼3 m and the diameter of the faceplate at maturity is about 80∼120 mm. The weight of receptacle and leaf is roughly 68 g. The diameter of plant, growth height and weight can help calculate the proper specifications of the spring.

**Figure 2.**
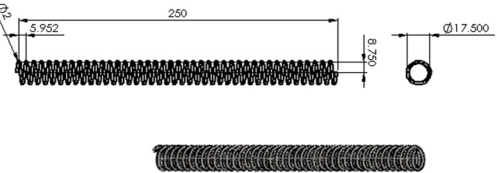
Spring specifications.

With regards to the setup of spring parameters, the outer diameter is set as 19.5 mm and length is 2.5 cm. Since the weight is 0.068 kg, the coefficient of spring is estimated as 0.07 Kgf/mm to prevent over buckling failure due to overweight or inadequate weight. Then, the spring diameter is set as *d* = 2 mm. These parameters are substituted into (1) to calculate spring turns *N_c_* = 39 shown as follows:

(1) $$N_{c}=\frac{{\mathrm{Gd}}^{4}}{{8D}_{m}{}^{3}k}$$

where G = 8,000 kg/mm^2^ denotes the modules of rigidity of wire, *D_m_* = (*D − d*) = 17.5 mm indicates the average diameter and *k* = 0.07 kgf/mm represents the modulus of elasticity of spring. *N_c_* is substituted into (2) to calculate spring pitch *P_itch_* = 6.4 mm:

(2) $$P_{\mathrm{itch}}=\frac{L+d}{N_{c}}$$

where *L* = 250 mm denotes length of spring. The outer diameter of spring, diameter, turns, and pitches can help determine the required spring. Figure 2 shows the specification of the purchased spring.

To realize the buckling degree the spring can withstand, we utilize the buckling theory to calculate the threshold for spring buckling to estimate the required power to produce buckling. The safety factor of spring buckling is estimated as 0.043. References for the detailed depiction are given in [26].

#### 2.2.3. Leafs and Petals

Leaf (petal) design requires plasticity and elasticity features. Thus, polydimethylsiloxane (PDMS) is utilized to construct the body of the leaves (petals) and a solid-state method is used to make them [27–29]. Because silicone belongs to the polymeric organosilicon compounds, it has good translucency and biocompatibility. Meanwhile, because it has a low Young’s modulus, the structure is high in structural flexibility and heat resistance, which is quite suitable for the material of leaves and petals.

**Figure 3.**
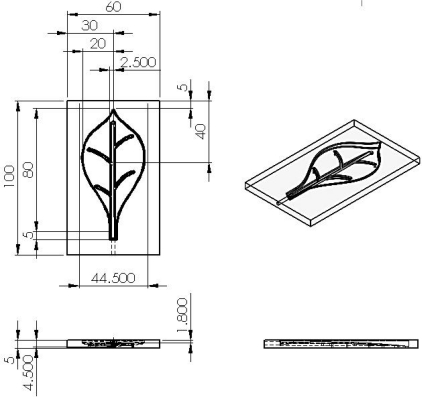
3D view of the leaves mold.

**Figure 4.**
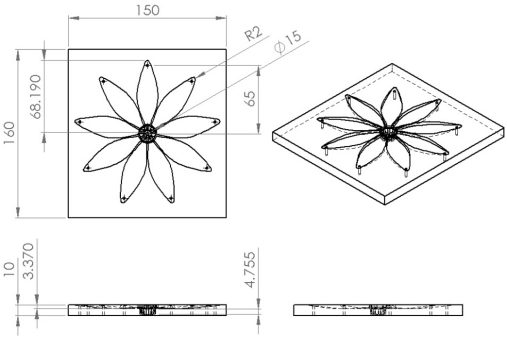
3D view of petals mold.

**Figure 5.**
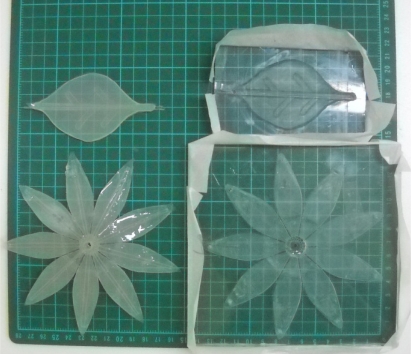
Leaf and petals of the template (right); finished product (left).

The PDMS material of leafs and petals can withstand the heat produced from the driving SMA. The silicone design process consists of six steps: (1) design of the mold for leaves and petals; (2) carved molding; (3) ratio blending of silicone and hardener; (4) mixing, pouring into template and pumping under vacuum; (5) placment under regular temperature for hardening; (6) template demolition and cutting of the silicone mold. Figures 3 and 4 depict the mold size of leaves and petals. Figure 5 shows the leaf and petal templates and the work after infusion molding.

#### 2.2.4. Leafs and Petals Swing Method

**Figure 6.**
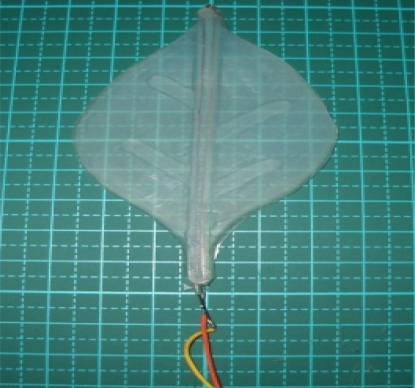
Silicone leaf with SMA.

Because the stems of leaves and petals are tinier, the spring cannot be utilized to swing in order to mimic a realistic effect. Thus, SMA is adopted to overcome this drawback. The SMA material serves as a blade branch. It can retain shape memory after heating. Thus, we utilize the characteristics of SMA to construct the leaves (petals), which results in the desired swing phenomenon. Firstly, the processed SMA is implemented in the silicone leaves. Second, the actuator that can control SMA is designed. The pulse width modulation (PWM) technique is employed to control the curvature of SMA and have the tip of leaf present displacement. Meanwhile, a thermal imager is adopted to analyze the generated temperature value of SMA under different voltages, which can help investigate the relation between the swings of leaves and petals of the bio-robot and the output voltage of the micro-controller. This can yield the optimal swing quality. Reference [30] describes the process in detailed. The final product is shown in Figure 6.

#### 2.2.5. Pot Incorporating Design

**Figure 7.**
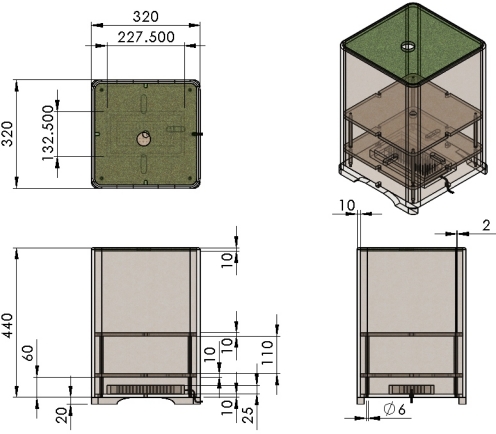
3D in view of pot.

The design specifications of the pot are presented in Figure 7, where the units are in mm. The pot consists of three layers. The bottom layer is used to place the controller and the peripheral modules of hardware system, *etc*. The middle layer is for the servomotor. The top layer is for the plant mechanism. The pot is covered with a clapboard and turf, which can add some sort of atmosphere.

### 2.3. Hardware Circuit Design

In this study, a self-designed embedded system is developed and the proposed system consists of DC motor control module, encoder, SMA actuator, light sensor modules, *etc*. The data is received from the multi-sensor modules, processed in micro-controller and then sent to the peripheral module to control the height and growth time of the bio-botanic robot.

#### 

##### System Architecture

The bio-botanic robot system consists of a BASIC Stamp module [31] and multi-sensor embedded board. Its detailed information is as follows:

(a) Power supply module.

The input voltage of each module in BRPS consists of 9 V, 6 V, and 5 V and the required power of each module is provided by three DC voltage regulator ICs, such as MC 7805, MC7806, and MC 7809, respectively. The power supply comes from a 12 V and 2.3 A battery.

(b) BASIC Stamp 2 (BS2) microcontroller module.

The BS2 microcontroller module is produced by the Parallax Company and it combines a micro-controller, EEPROM memory chip, serial transmission interface, voltage regulator IC, oscillator and noise filter, *etc*. [31]. What is special about the chip is that it has an inbuilt PBASIC interpreter, which can allow the user to rapidly develop the required control application programs. Thus, the BS2 module serves as the sub-core control unit of BRPS to control the signal transmission between each module.

(c) USB communication modules.

Bi-directional serial communication between BS2 and PC.

(d) Inter-Integrated Circuit (I2C) communication modules.

The BRPS multi-staged fuzzy control technique [25] is implemented via a PIC microcontroller. To maintain smooth communication between the PIC and the BS2 microcontroller so as to have the values of environmental factors collected by the BS2 microcontroller sent to the PIC one to determine the growth mode and have the decision value sent back to the BS2 microcontroller to effect the corresponding output control, the I2C communication interface serves as the bridge between the PCI and the BS2 micro-controllers. The greatest feature of the I2C module lies in the use of the communication clock pin and data pin of the PIC micro-controller and the connection of one pull-up resistance to these two signal pins to accomplish the I2C serial data communication interface.

(e) Light sensor module.

It consists of a photosensitive resister and IC 555 oscillator. The goal of these modules is to detect the strength of light and to convert the light power into the signal so that the module can identify it;

(f) Temperature and humidity sensing module.

Water and temperature are the environmental factors of the proposed system and this module adopts the module designed by the Parallax Company. The model of this module is SHT11 [32]; its range of temperature is between −40 °C to +123.8 °C, precision is ±0.5 °C, humidity is between 0 and 100%RH and precision is ±3.5%RH.

(g) DC motor control module.

The design purpose of this module is to effectively and precisely adjust the growth rate of the robot. This research designs a DC motor control module to control the motor. Firstly, the motor is defined based on the growth time of plants. This research defines the optimal growth time as 120 seconds and growth height as 30 cm. Thus, the longest rotation time of motor is 2 minutes. The growth height is regarded as the circumference length of the driving wheel and the diameter of driving wheel is calculated as 30/π = 9.549 cm. To reduce the space occupied by the transmission, the diameter of the driving wheel is shortened and a commercially available driving wheel is chosen, whose diameter is 2.4 cm. That is, the motor rotates four times to reach growth height of 30 cm. Thus, the rotations per minute (rpm) are calculated as 2. Besides, the growth rate of plant in each stage differs, where the greatest difference lies in the second and the third stage. In that stage, the plant grows 1 cm each second. In this research, the motor purchase is based on the criteria of the second and third stage. Thus, the driving wheel only needs to rotate 0.133 times in order to grow 1 cm, which is calculated as 8 rpm for the motor. As a result, we can select a driving wheel with a motor speed as 8 rpm and 2.4 cm diameter for the drive component of the rising mechanism in this system. The DC motor control module is powered by a TA7291P DC driver to achieve the functions of speed control, rotation, stop and emergency stop [33]. The TA7291P is composed of one H bridge driver circuit and a compensation circuit. It is equipped with a maximum voltage 25 V and a maximum output current of 2 A. In this control module, the output pin of the BASIC stamp microcontroller is employed to transmit a PWM signal to adjust the reference pin (pin 4) of the TA7291P chip to control motor speed and to give the high and low voltage to pin 5 and pin 6 of TA7291P to change work mode.

(h) Motor encoder module.

The module is completed by using SG-206 photo-interrupters [34]. The main function of the motor encoder module is to estimate the growth height of the robot.

(i) SMA actuator.

The module is to derive the leaf and petals of the swing.

(j) Servo motor control module.

The servo motor employed in this system is used to control the degree of blending of stems. One point of the turntable of the servo motor is illustrated with a fixed rope and on the other point of the rope is fixed on the spring point. The rope is moved by the operation of the servo motor so as to represent the curvature of the spring. The motor control module uses what is already on the basic stamp development board. In addition, a light emitting diode circuit is utilized to indicate whether the communication between Basic stamp microcontroller and servo motor is smooth.

### 2.4. Software Programming Design

The software system consists of a personal computer (PC) side and BASIC stamp controller. In this research, the a PC program is adopted to initialize and acquire the related parameters of the robot. BASIC Stamp utilizes Basic stamp editor v2.5 developed by Parallax as the program development tool, which has one specific programming language interpreter called PBASIC. Thus, BASIC Stamp controller is applied to the experimental board of the bio-botanic robot to control data transmission and reception of each module. Besides, a Fuzzy Knowledge Base (FKB) is inserted in the memory to execute programs [25]. The following will describe the program design process of each function in the system.

#### 2.4.1. The Flow Chart of Main Program

**Figure 8.**
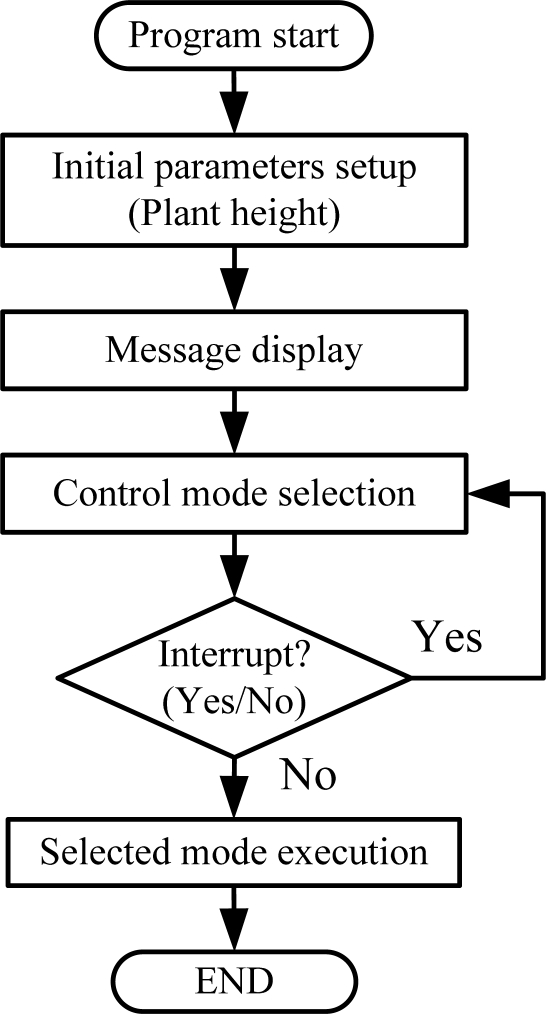
Flow chart of the main program.

Figure 8 depicts the flow chart of the main program. When the system power is on, the system restarts, initializes, and the internal parameter is to be setup within the controller. Firstly, the parameters of plant height, amount of light, temperature, humidity, and the growth stage are initialized. The status information of the system is to be displayed on the screen of PC and then the system enters standby mode.

#### 2.4.2. The Control Command

**Figure 9.**
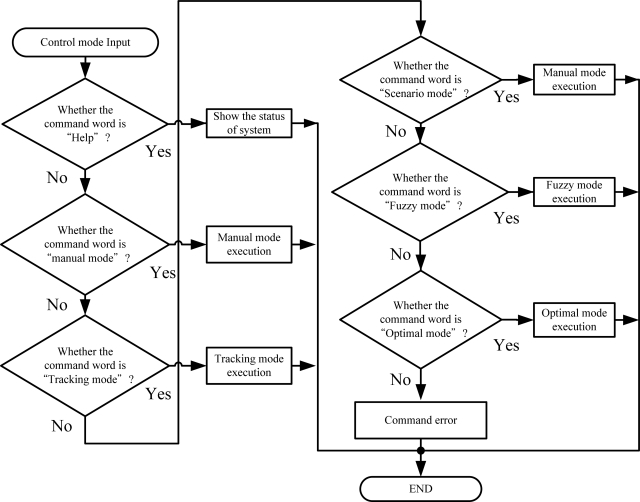
Sub-program for mode selection.

Two types of command are executed in the bio-botanic robot. The first one is to select growth mode, which includes the optimal growth function, fuzzy growth function, and manual function. The second type is scenario mode that consists of light tracking function and light scenario function. Figure 9 depicts the flow chart of the control process.

**Figure 10.**
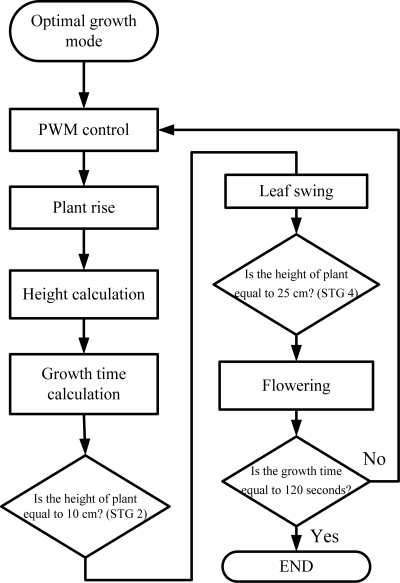
Flow chart of the optimal growth mode program.

Each function is demonstrated in the following paragraphs:

(A) Growth mode:

Set_ Best: To perform the optimal growth function. If the system is switched to this function, the robot will grow in full accordance with the desired parameters. Firstly, the speed control value of motor is calculated based on different growth rate in each stage. Next, the driving wheel drives the spring to move the stem of the plant upwards. Meanwhile, the encoder module begins to calculate the growth height. Figure 10 demonstrates the flow chart of the optimal growth mode program.

**Figure 11.**
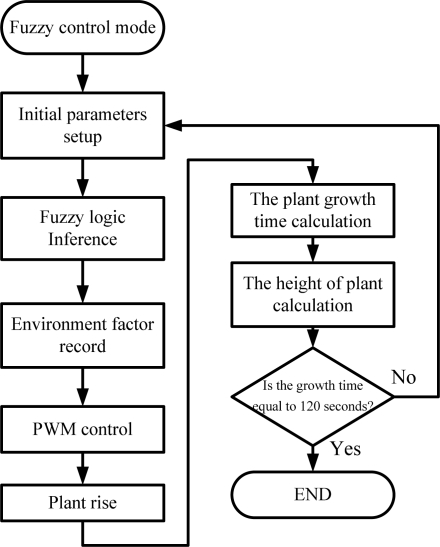
Flow chart of the fuzzy control mode program.

Set_FC: To perform the fuzzy growth function. If this function is in operation, the growth rule of robot is established by the expert knowledge data. The rule data can adjust the amounts of environmental factors and meet the desired values. Firstly, the initial parameters are sent to the fuzzy controller. After the fuzzy logic process, the output from the fuzzy controller is then stored in the memory on the BASIC Stamp board. Meanwhile, the output value of fuzzy controller is then sent back to the input of fuzzy system. After several iteration processes, the control value is sent to the BRPS embedded board to control the motor speed and rotatation direction. At the same time, the encoder records the times and growth rate of the robot. When the height of plant reaches the wither stage, the robot stops growing. Figure 11 shows the flow chart of program of fuzzy-based growth mode.

**Figure 12.**
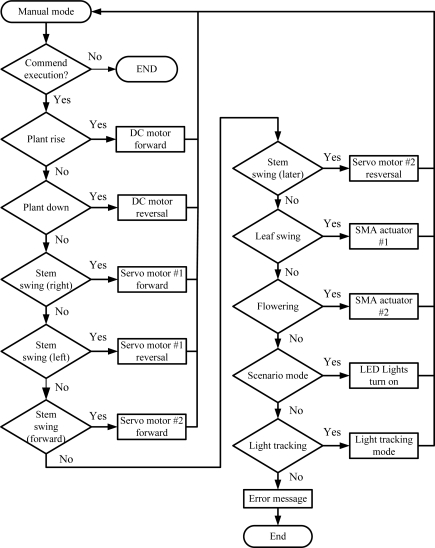
Flow chart of the manual mode program.

Jump_Manual: To execute the manual function. The main purpose of this function is to prevent the occurrence of emergencies. Meanwhile, the function is also used to test each growth modes for the bio-botanic robot. Figure 12 depicts the flow chart of program of “Jump_Manual”.

(B) Scenario mode:

Track_Sun: To start the light tracking function. The output of the maximum value from the four light sensor modules is selected through a photosensitive resistor. The controller is used to start the servo motor and the motor location corresponds to the maximum output of the resistors. Finally, the motor pulls the spring at the top of the fixed wires and the stem of robot is bent toward the direction of maximum light intensity swing.

Star_Light: To start the light scenario function. Once the function is started, the system firstly identifies the status of the robot and then decides whether to turn on the light within the robot.

## 3. Experimental Results

**Figure 13.**
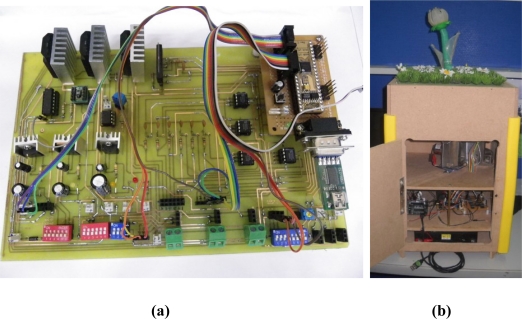
Multisensor-based bio-botanic robot system. (**a**) Proposed embedded board; (**b**) Experimental equipment.

This chapter demonstrates the experiment test results of our bio-botanic robot. Figure 13 shows the self-designed embedded board and experimental equipment. The embedded development board integrates the required modules for BRPS to execute each function, which includes power supply module, main-controller, sub-controller, random access memory (RAM)/read only memory (ROM) modules, DC motor driver module, motor encoder module, SMA driver modules, temperature/humidity sensors modules, light sensors modules, and universal serial bus (USB) communication module. The BRPS consists of plant base, embedded board, micro-controller, and is contained within the pot.

**Figure 14.**
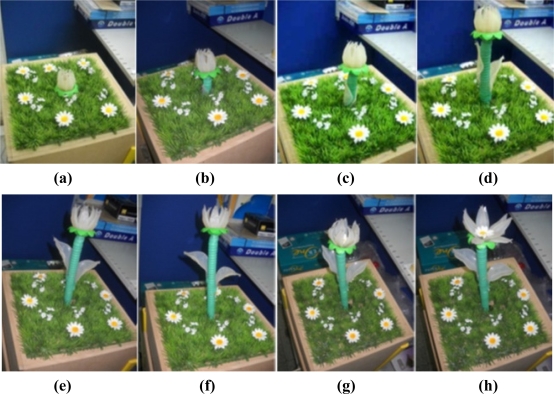
Different growth stages of the bio-botanic system.

**Figure 15.**
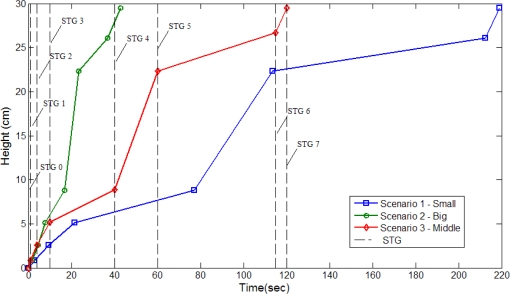
Curve of growth rate (simulated).

**Figure 16.**
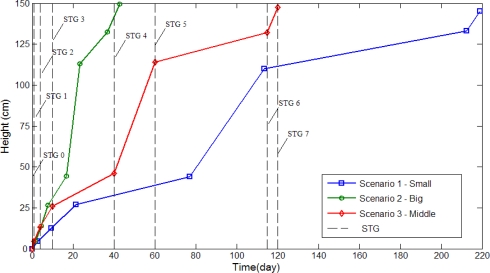
Curve of growth rate (experimental).

**Figure 17.**
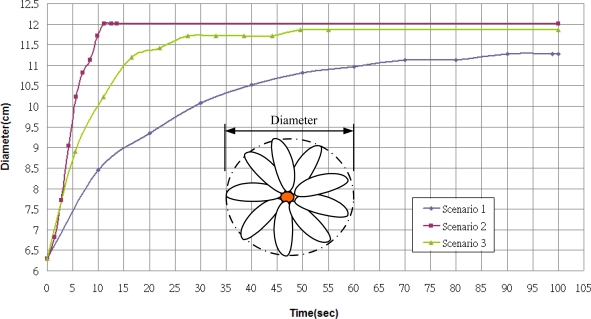
Curve of flowering (STG6).

Three different growth modes are considered in these experiments. Different parameter settings allow observation of changes in growth rate and environment factors are regulated by the fuzzy controllers. Figure 14 the depicts status of plant growth, which is divided into eight stages shown as follows (first scenario): (a) STG0 stage; (b) STG1 stage; (c) STG2 stage; (d) STG3 stage; (e) STG4 stage; (f) STG5 stage; (g) STG6 stage; (h) STG7 stage. Figures 15 and 16 illustrate the growth curve of BRPS and real plants. The two figures show that the experiment of growth height under three different environmental factors almost resembles the growth process of real plants. It shows that the proposed BRPS can mimic the plant growth based on different environmental factors. Figure 17 demonstrates the flowering stage (STG6) of the robot for three scenarios.

## 4. Conclusions

The design concept of a bio-botanic robot is proposed in this paper. The robot receives information from an expert database and multi-layer fuzzy controllers, which are utilized to complete the plant growth, light tracking, *etc*. The magnitudes of environmental factors are implemented and quantized using MEMs technology and all of the sensor modules are integrated in a self-designed embedded board. Meanwhile, the implementation of a fuzzy control system also allows BRPS to have autonomous growth behavior. The system presents the novel concept, which could be used for a future automated cultivation system. In addition, the use of the scenario functions within the robot can also serve as a teaching tool and material.

## Supplementary Information
