# Supplementary material for: Development of a Multisensor-Based Bio-Botanic Robot and Its Implementation Using a Self-Designed Embedded Board
Source: Sensors (Basel). 2011 Dec 13;11(12):11629–48. doi: 10.3390/s111211629 (PMC3252001; doi:10.3390/s111211629)
Supplement: Supplementary file 1 [file sensors-11-11629-s001.pdf]

Supplementary Information

Development of Multisensor-Based Bio-Botanic Robot and Its Implementation Using Self-Designed Embedded Board

Chung-Liang Chang *, Ming-Fong Sie and Jin-Long Shie

Department of Biomechatronics Engineering, National Pingtung University of Science and Technology, No. 1 Shuefu Road, Neipu, Pingtung County 91201, Taiwan;
E-Mails: m9844014@mail.npust.edu.tw (M.-F.S.); j357753k@yahoo.com.tw (J.-L.S.)

* Author to whom correspondence should be addressed; E-Mail: chungliang@mail.npust.edu.tw; Tel.: +886-8-770-3202 ext. 7586; Fax: +886-8-774-0420.

Received: 1 November 2011; in revised form: 25 November 2011 / Accepted: 5 December 2011 / Published: 13 December 2011

Figure A-1. Circuit diagram of power module for BPRS.

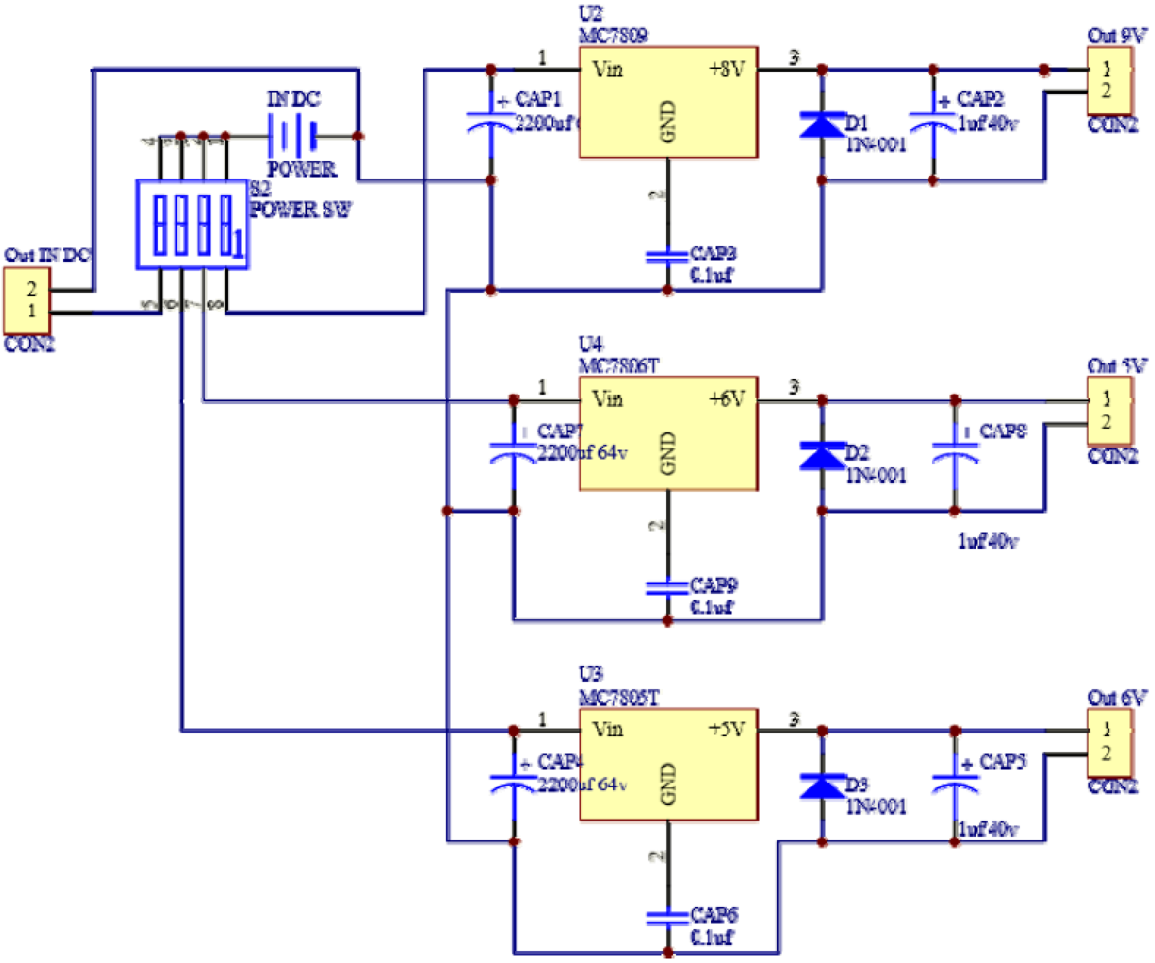

Figure A-2. Circuit of light sensors modules.

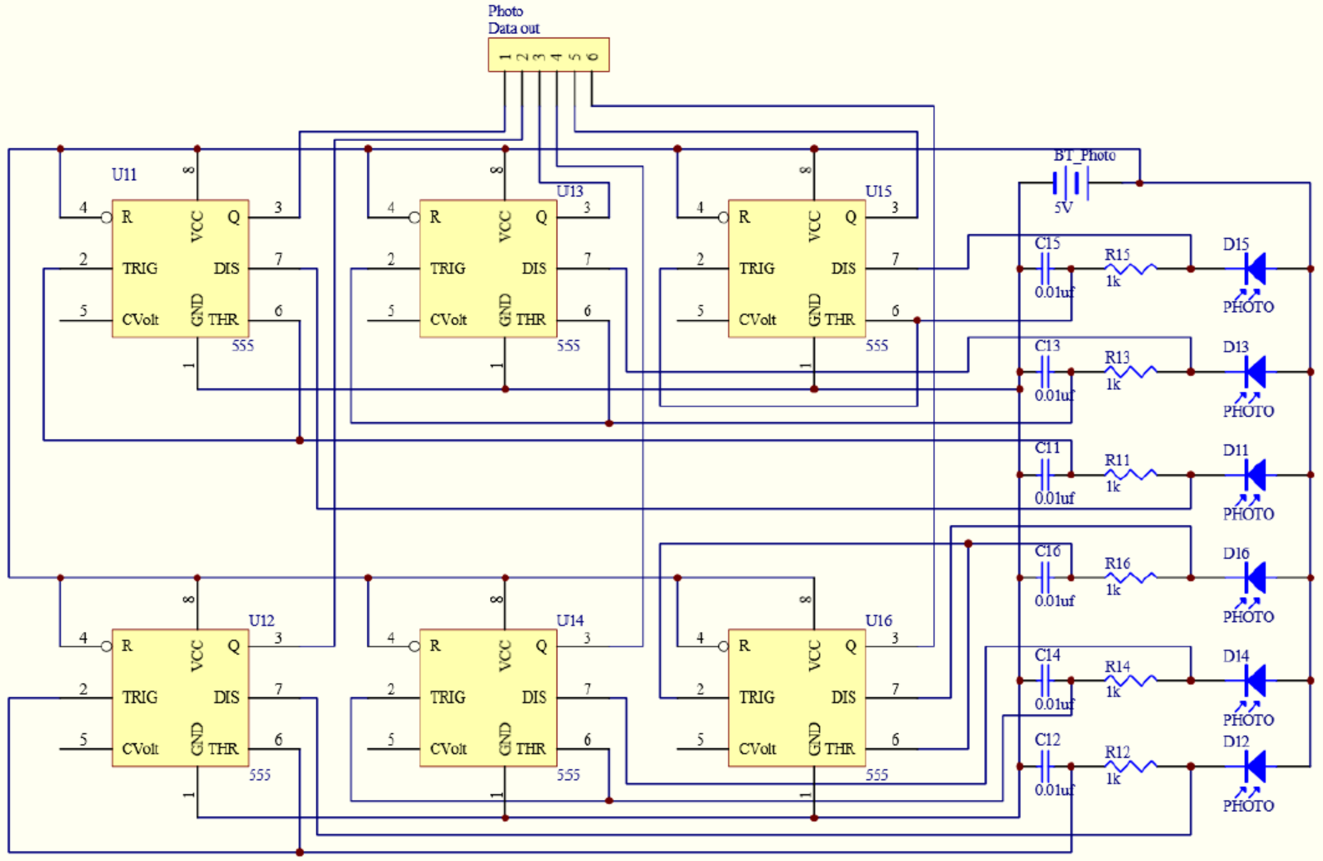

Figure A-3. SHT11 temperature/humidity circuits.

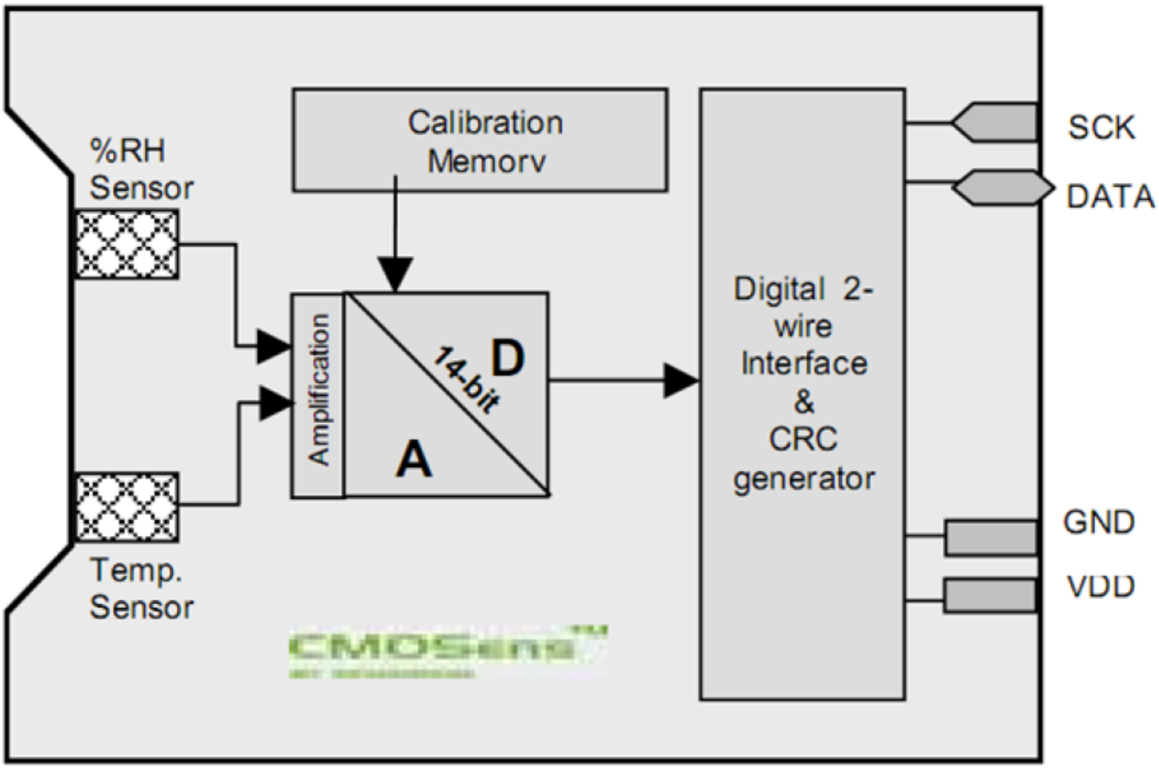

Figure A-4. BASIC stamp controller via USB communication modules.

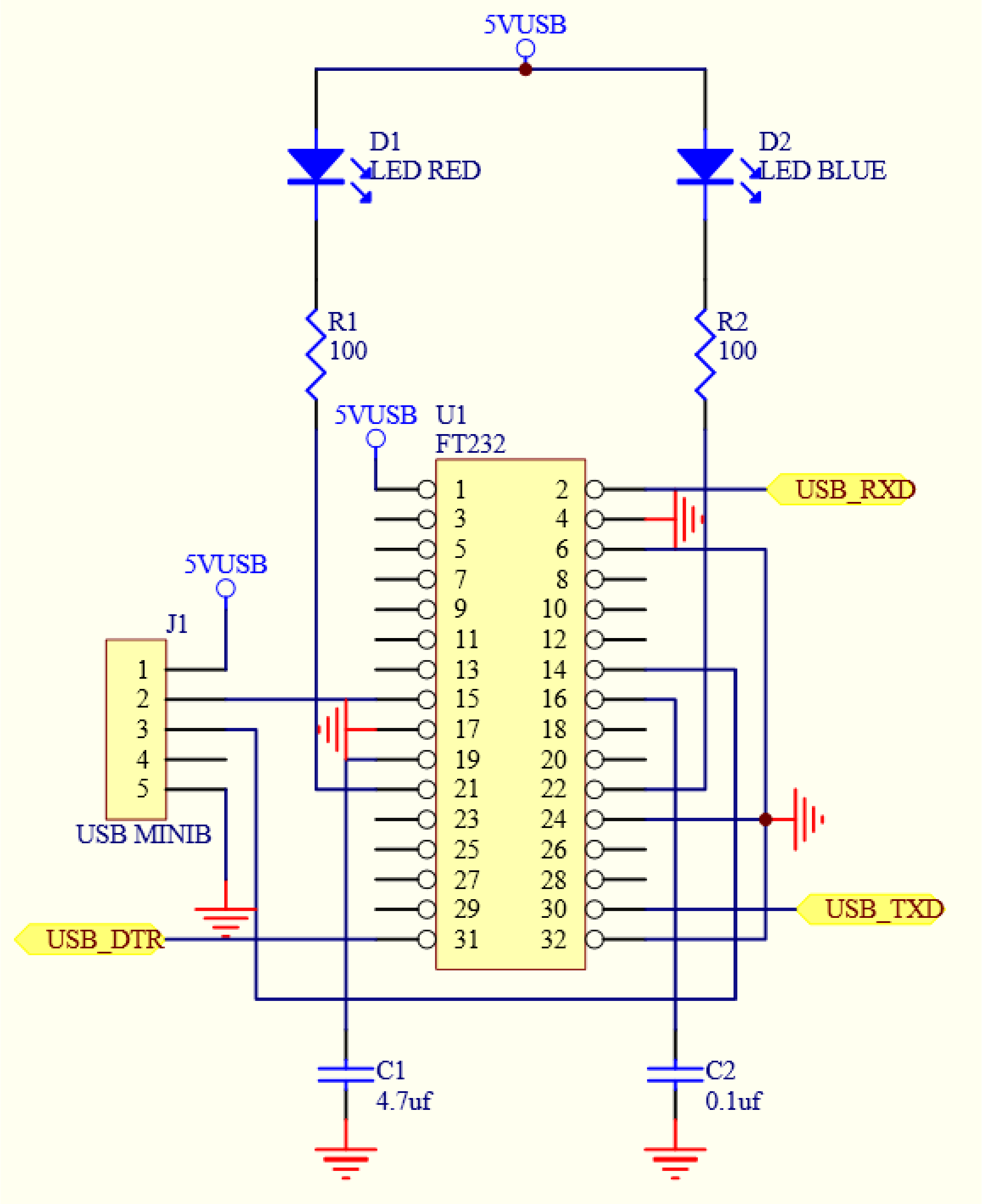

Figure A-5. Circuit of DC motor module.

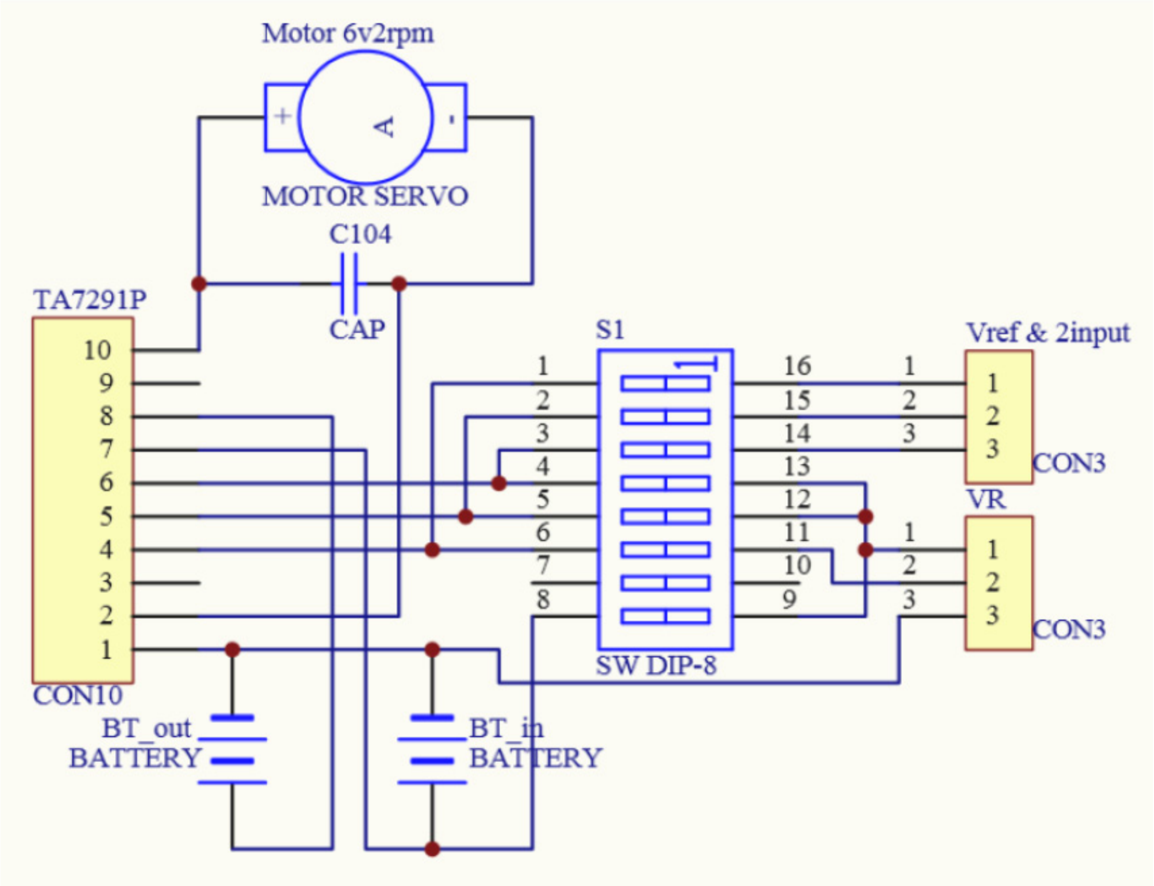

Figure A-6. Circuit of SMA modules.

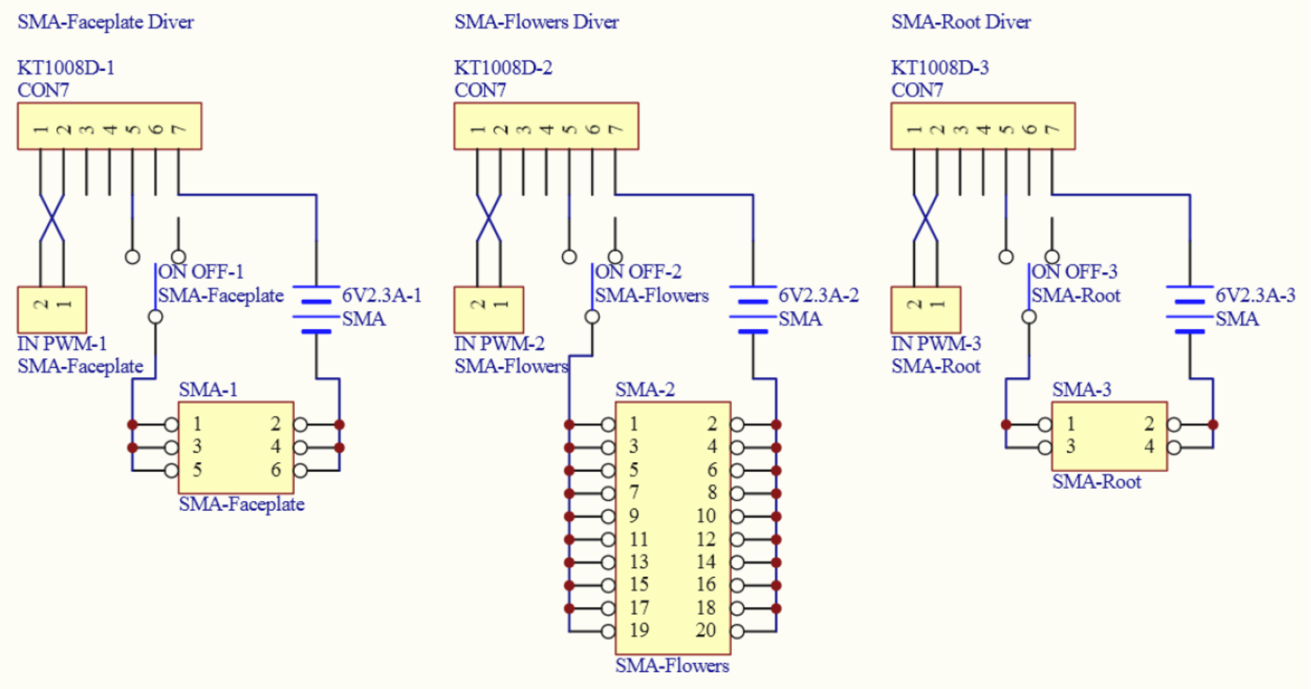

Figure A-7. Circuit of KT1008D SSR.

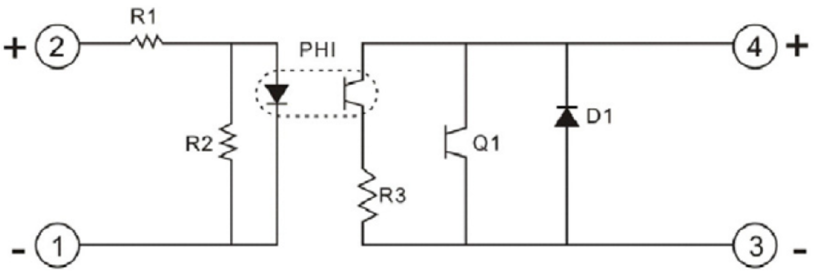

Figure A-8. Layout diagram of BPRS embedded board (Ver.1.0).

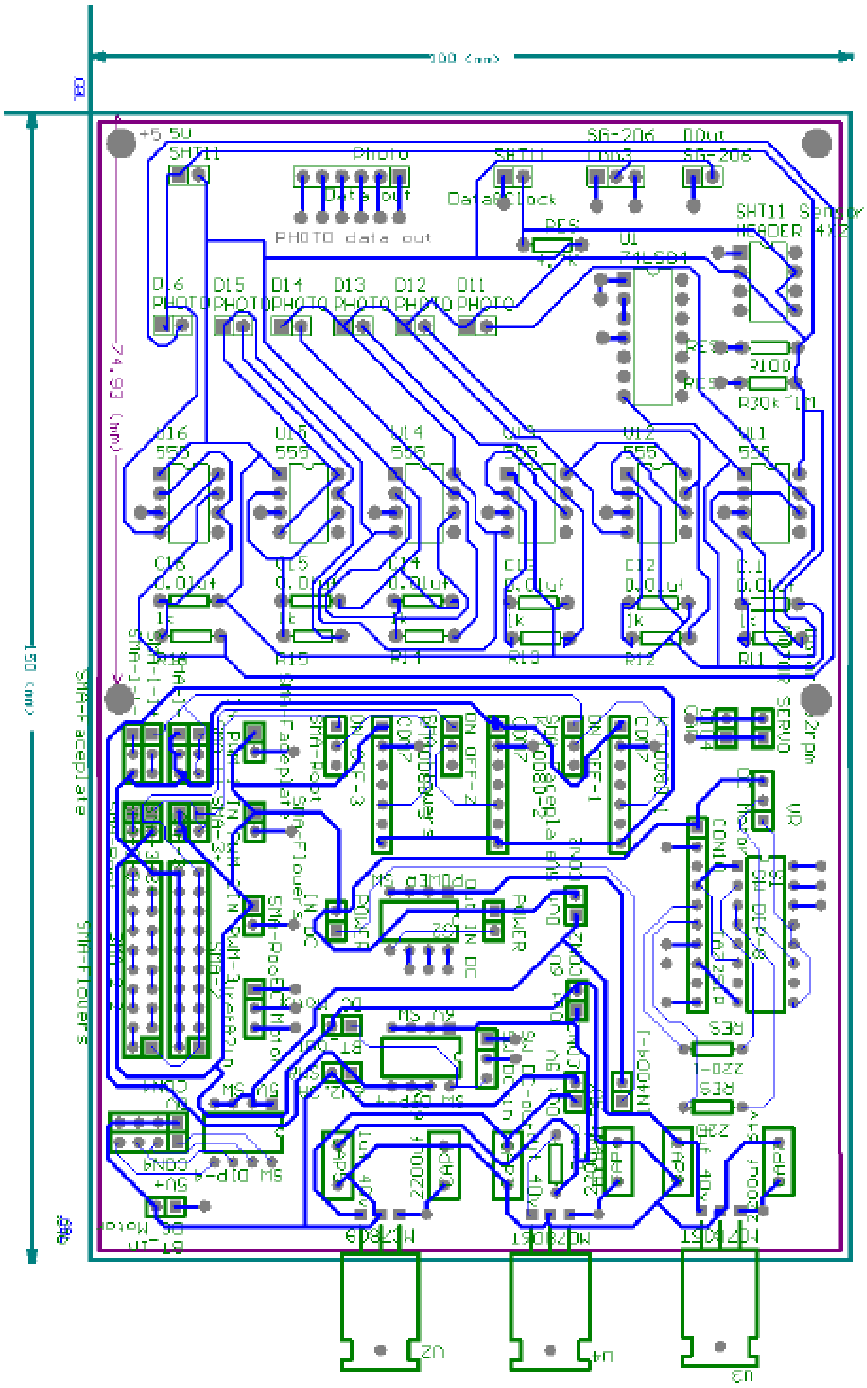

Figure A-9. Circuit diagram of BPRS embedded board (Ver.1.0).

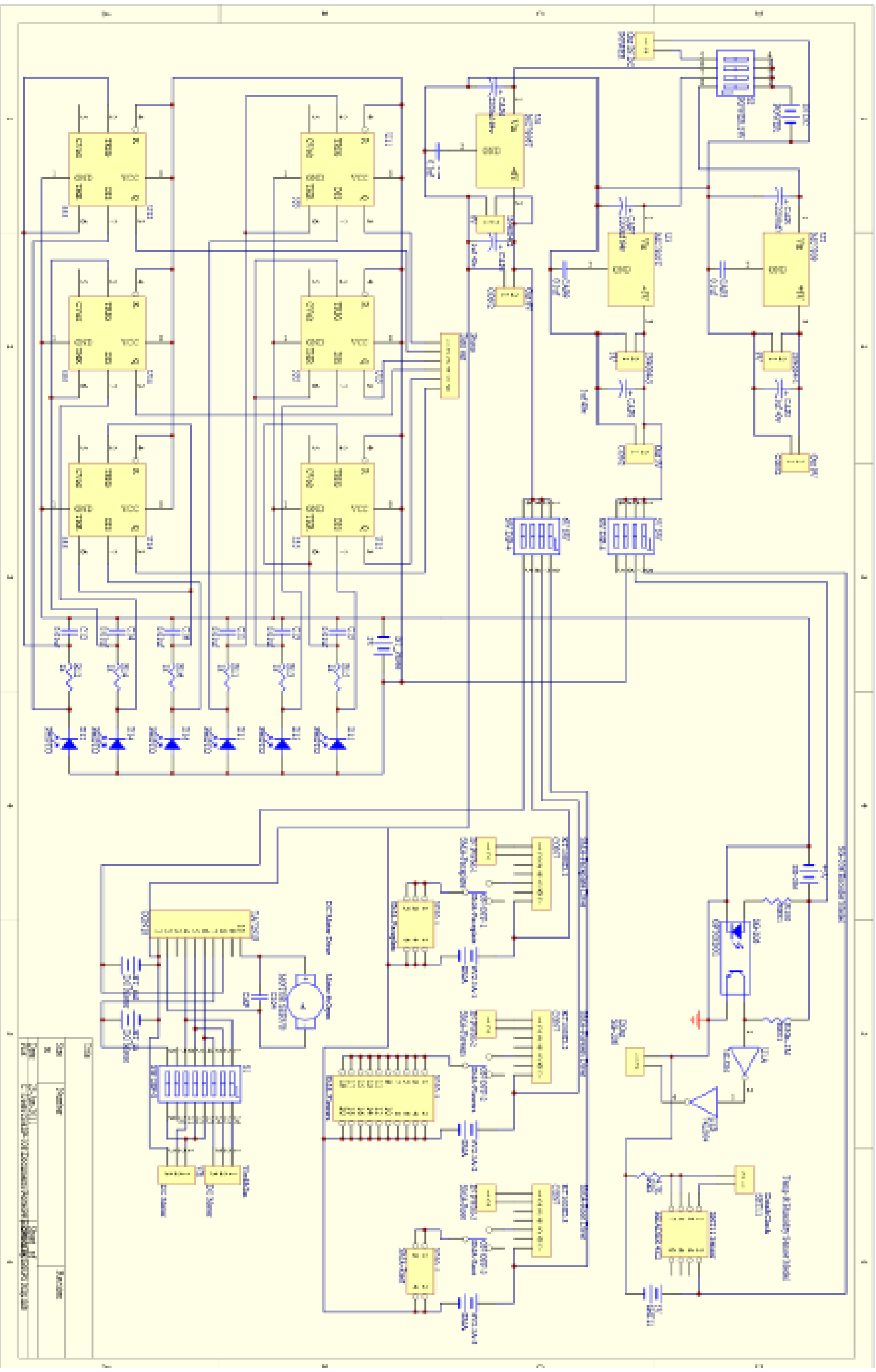

© 2011 by the authors; licensee MDPI, Basel, Switzerland. This article is an open access article distributed under the terms and conditions of the Creative Commons Attribution license (http://creativecommons.org/licenses/by/3.0/).
